# Acute kidney injury associated with rhabdomyolysis after coronary artery bypass graft: a case report and review of the literatures

**DOI:** 10.1186/1756-0500-7-152

**Published:** 2014-03-17

**Authors:** Suraj Sudarsanan, Amr S Omar, Rasheed A Pattath, Abdulwahid Al Mulla

**Affiliations:** 1 Department of Cardiothoracic Surgery/Cardiac Anaesthesia & ICU Section, Heart Hospital, Hamad Medical Corporation, Doha (PO: 3050), Qatar; 2 Department of Critical Care Medicine, Beni Suef, Egypt

**Keywords:** Acute kidney injury, Rhabdomyolysis, Coronary artery bypasses graft, Prolonged surgery

## Abstract

**Background:**

Post-operative rhabdomyolysis is a well-known complication, especially after bariatric and orthopaedic surgeries. There are few published reports of rhabdomyolysis following cardiac surgery. Acute kidney injury had been distinguished as a serious complication of cardiac surgery. We report a case of 55-years-old male patient who developed rhabdomyolysis precipitated acute kidney injury after coronary artery bypass graft.

**Case presentation:**

The patient underwent urgent coronary artery bypass graft surgery, with a long duration of surgery due to technical difficulty during grafting. He developed rhabdomyolysis induced acute kidney injury necessitating hemodialysis. The patient in turn developed heart failure, which along with acute kidney injury lead to prolonged ventilation. There was supervening sepsis with prolonged intensive care unity stay and eventually prolonged hospitalization. The peak creatine kinase level was 39000 IU/mL and peak myoglobin was 40000 ng/ml. Reviewing the patient, surgery was prolonged due to technical difficulties encountered during grafting, leading to rhabdomyolysis induced acute kidney injury. The pre-operative use of statins by the patient could also have contributed to the development of rhabdomyolysis. He developed post-operative right heart failure and sepsis. The patient’s renal function gradually improved over 4 week’s duration. Favorable outcome could be achieved but after prolonged course of renal replacement therapy in the form of hemodialysis.

**Conclusion:**

Prolonged duration of surgery is a well-recognized risk factor in the development of rhabdomyolysis. Early recognition of rhabdomyolysis induced acute kidney injury is important in reducing the post-operative morbidity and mortality in patients. A protocol based approach could be applied for early recognition and management.

## Background

Cardiovascular diseases is identified as a leading cause of mortality all over the world according to the recent data by the World Health Organization (WHO), the mortality included 51% due to strokes and 45% due to coronary heart disease [1]. Rhabdomyolysis (RML) is a dissolution of skeletal muscles that produces a nonspecific clinical syndrome causing extravasation of toxic intracellular contents from the myocytes into the circulatory system [2]. This destruction leads to electrolyte disturbances, hypovolemia, metabolic acidosis, coagulopathies and myoglobinuric renal failure. This abnormality is associated with more than 100 seemingly unrelated disorders, including direct muscle injury (crush injury syndrome), muscle ischemia, excessive physical exertion, temperature extremes, infections, drugs, toxins, venoms, and endocrine disorders, among others. Although RML was initially recognized solely as a posttraumatic sequela, nontraumatic causes are now estimated to be more frequent than traumatic causes [3].

Rhabdomyolysis with subsequent myoglobinuria as a cause of acute renal insufficiency was first described by Meyer-Betz in 1911. Compression-induced rhabdomyolysis has been reported in connection with several operations, in particular in certain positions for surgery. In non-traumatic patients after elective surgery RML usually occurs as a result of compression because of unsuitable positioning or tourniquet use, but there are other contributing factors that should be considered [4]. Recently it has been recognized that myoglobinemia-induced acute kidney injury (AKI) may play a crucial role in surgical settings, especially with urologic [5] and thoraco-abdominal aortic surgery [6]. In bariatric surgery, RML is considered a consequence of the high pressure on the muscles on the operating table [7]. Extreme positions such as that for lithotomy may lead to RML even in non-obese patients [8]. In laparoscopic bariatric surgery, gluteal and back muscles are at danger because of the patient’s position [9,10]. Perioperative myocardial injury cannot totally explain the occurrence of increased myoglobinemia. Skeletal muscle breakdown and necrosis play an important role in determining increased myoglobin concentration after coronary artery bypass grafting (CABG) [11].

Rhabdomyolysis during or after cardiopulmonary bypass (CPB) is not very common [12]. Preoperative medication seems to be causative in certain cases. A correlation of RML and direct femoral artery cannulation, arteriopathy, prolonged extracorporal circulation, low cardiac output syndrome, and continuous intravenous infusion of epinephrine could be shown. There have not been many published studies of RML in adult patients undergoing CPB [13]. We report a case of a patient who developed RML with acute renal injury following CABG.

## Case presentation

A fifty-six-year-old male, weighing 60 kg and height 160 cm, chronic smoker (40 pack years) quit four months before admission, with history of occasional alcohol intake. He was a known case of hypertension and coronary artery disease (CAD) of 10 years duration, but on irregular treatment. Patient presented on 03/05/2012 with unstable angina, underwent urgent coronary angiogram, which revealed 100% stenosis of right coronary artery (RCA) and 80% stenosis of left anterior descending (LAD) branch. An attempt at percutaneous coronary angioplasty (PTCA) to RCA failed and patient was referred for surgical revascularization. Preoperative medications included acetyl salicylic acid, metopralol, amlodipine, rosuvastatin, and ivabradin. Clopidogrel was discontinued 10 days prior to surgery. All biochemical investigations prior to surgery were normal, including renal and liver function tests.

Patient was premedicated with oral lorazepam (2 mg) on the night before surgery and morphine 5 mg intramuscular with metoclopramide 10 mg intramuscular on the morning of surgery just before transferring to operating room. Anesthesia was induced with intravenous boluses of fentanyl, midazolam, etomidate and cisatracurium, airway was secured with an 8.5 mm internal diameter (ID) endotracheal tube. Standard monitoring including arterial blood pressure (20 G in right radial artery), central venous pressure (8.5 Fr. right internal jugular vein), 5 lead electrocardiogram (ECG), pulse oximetry (SPO_2_), end tidal carbon dioxide (ETCO2), end tidal concentration measurement of inhalational agents were instituted. Anesthesia was maintained with intravenous infusions of propofol (60 mg/hour), fentanyl (250 mcg/hour), cisatracurium (3 mcg/kg/minute) and sevoflourane at 1 minimum alveolar concentration (MAC) as inhalational agent. After harvesting the left internal mammary artery (LIMA) and right internal mammary artery (RIMA) and systemic heparinisation with 400 IU/kg, the patient was put on cardiopulmonary bypass (CPB) after securing an activated clotting time (ACT) of 480 seconds.

**Figure 1:**
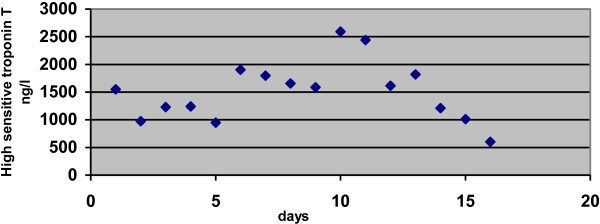
High sensitive troponin T through the hospital stay.

The LIMA graft was anastomosed to LAD. The anastomosis of RIMA to RCA was beset with technical difficulties necessitating repeated return to CPB thrice to redo the graft as there was poor flow, and eventually a saphenous vein graft was used to anastomose RCA. Total CPB time was 250 minutes and the aortic cross clamp time was 55 minutes (Figure 1). The patient was weaned off CPB with modest doses of adrenaline and noradrenaline infusions. Patient received 2 units of packed red blood cells (PRBC), 6 units of platelets and 2 units of fresh frozen plasma (FFP), directed by thromboelastography (TEG) during the procedure. The total duration of surgery was nine hours. The patient was shifted to the cardiothoracic surgery intensive care unit (CTICU) for recovery.

The patient remained hemodynamically stable in the CTICU. His laboratory showed increase in serum creatinine to 185 micromol/L, had persistently elevated potassium levels (corrected by glucose insulin boluses) and mild lactic metabolic acidosis. Patient was extubated around midnight on day zero as he maintained stable hemodynamics with minimal chest drains. He remained in the CTICU for four days, during which time his serum creatinine remained elevated, but urine output was maintained at >1 mL/kg/hour, albeit with reddish discoloration. The patient’s serum potassium remained elevated and calcium resonium was started as per nephrologist’s advice. The patient’s creatine kinase (CK) remained elevated from zero post-operative day and a diagnosis of RML was made. At the same time there was mild derangement in liver function tests. Patient was transferred to surgical step-down unit (SSDU) on the fourth post-operative day.

The patient’s course in the SSDU was complicated by a progressive rise in serum creatinine, CK, myoglobin, and liver enzymes. There was progressive decline in urine output and furosemide infusion was instituted, with little improvement. At the same time the patient became septic as evidenced by elevated levels of procalcitonin and white blood cell (WBC) counts and was started on broad spectrum antibiotics for suspected nosocomial infection. The patient was shifted back to the CTICU on the eighth post-operative day as his general condition had deteriorated with fluid overload, sepsis and AKI.

**Figure 2:**
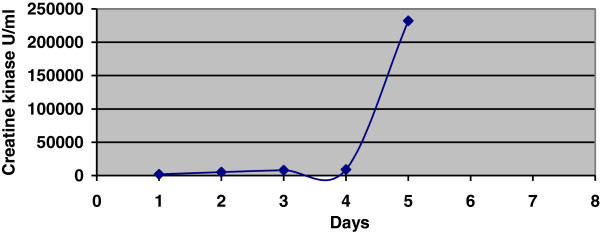
Creatine kinase progression till readmission in the intensive care unit.

On readmission to the CTICU his serum creatinine was 460 micromol/L, CK was 23321 u/L (Figure 2), and serum myoglobin was 40228 ng/ml, with significant elevation in liver and cardiac enzymes. He became anuric and had severe lactic metabolic acidosis on the day of readmission to CTICU, and hemodialysis was instituted. Transthoracic echocardiogram (TTE) was done on the 11th post-operative day revealed severe right ventricular dysfunction with normal left ventricular function (LVF). The patient’s general condition worsened progressively with drowsiness, hypoxia, hypotension, and evidence of low cardiac output, and he was electively intubated and put on mechanical ventilation on the 11th post-operative day. The patient was kept sedated with infusions of remifentanil (0.05-0.1 mcg/kg/min) and propofol (50–70 mg/hour). Hemodynamics was maintained with infusions of dobutamine (5–10 mcg/kg/min) and noradrenaline (0.05-0.1 mcg/kg/min) and vasopressin (2–4 u/hour).

On the 12th post-operative day the patient developed abdominal distension and melena, his serum biochemistry revealing deranged coagulation profile, liver function tests (LFTs), worsening renal parameters and increased markers of sepsis. He was kept sedated and ventilated from the 11th to the 18th post-operative day. The patient developed generalized edema and intra-abdominal pressure was elevated. CT scan of the abdomen revealed moderate ascites and diffuse liver surface nodularity. Klebsiella and Enterobactor faecalis were isolated from the patient’s urine during this period. The patient underwent regular hemodialysis with gradual restoration of renal function and was extubated after eight days of sedation and ventilation on the 18th post-operative day. He was transferred out of the CTICU to SSDU after 14 days of stay on the 22nd post-operative day. The patient underwent regular nephrology follow-up with frequent hemodialysis until his renal function recovered after a further two weeks. He was discharged from the hospital in another week and has been followed up regularly. He had recovered from his heart failure, as evidenced by transthoracic echocardiography (TTE) done prior to discharge. His total hospital stay was 56 days, of which 46 had been post-surgery.

## Discussion

**Figure 3:**
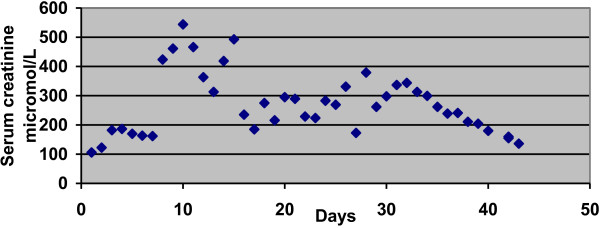
Serum creatinine progression through the hospital stay.

Rhabdomyolysis after CABG is defined as having a CK level more than 2500 u/L [14]. Use of myoglobin is confirmatory [15]. According to the consensus definition proposed by the Acute Kidney Injury Network, AKI was defined as an abrupt (within 48 hours) reduction in kidney function defined as an absolute increase in serum creatinine concentration of 0.3 mg/dL or greater (26.4 mmol/L) or a percentage increase of 50% or greater (1.5-fold from baseline). We did not use urine output in defining AKI (Figure 3) [14]. Postoperative low cardiac output syndrome (LOS) was defined as the need for a postoperative intra-aortic balloon pump or inotropic support for longer than 30 minutes in the intensive care unit to maintain systolic blood pressure at greater than 90 mm Hg [16]. Perioperative myocardial injury is diagnosed when the troponin I (cTnI) concentration at 1 hour after aortic declamping was greater than 0.92 ng/mL [17]. The European system for cardiac operative risk evaluation (Euro-SCORE) [18] was used to assess differences in patients’ risk profiles.

We are reporting a patient who presents with plentiful risk factors for RML development after surgical intervention. Rhabdomyolysis is a known cause of morbidity and mortality. It had been described on many occasions in trauma and after surgeries, but the incidence of RML after cardiac surgeries is quite variable, and had been described only in individual case report. Some authors report a direct relation between AKI and RML, while others report a 19% incidence of RML after CABG [11].

Multiple factors have been claimed to be the causes of RML after cardiac surgery. A correlation of RML and direct femoral artery cannulation, [19] arteriopathy, prolonged extracorporal circulation, low cardiac output syndrome, and continuous intravenous infusion of epinephrine has been described. Furthermore, diabetes mellitus, advanced age, and preexisting renal impairment have been mentioned to be contributory factors [20,21]. Rhabdomyolysis has also been reported in patients with pressure necrosis due to incorrect positioning during surgery [22,23] and in patients with an intra-aortic balloon pump [19].

In this case, most of these causative factors could be excluded, because they did not pertain to our patient. Prolonged surgeries, extreme surgical positions, American society of anesthesiologists (ASA) physical status III–IV, and the presence of diabetes or hypertension have also been identified as added factors could precipitate development of RML [24]. The operating room has been considered as a favorable environment for RML occurrence because of unusual positions and areas with increased pressures under anesthesia. Sustained high muscle pressure induces muscle ischemia, direct injury to sarcolemma, disruption of sodium–potassium pump, electrolyte imbalance, and failure of energy supply to the muscle fiber [25].

Our patient was 56 years old, he was not known to be diabetic, he had normal preoperative renal functions, he was ASA class IV, his Euro-SCORE was 3, and he had long surgery (about nine hours) as well as long bypass time (250 minutes).

Statins were started for treatment of his hyperlipdemia 20 days prior to admission. Some authors reckon that lipid-lowering medications are associated with a high risk of perioperative mortality; moreover, they suggest discontinuing the intake prior to surgical interventions [26]. However numerous studies have demonstrated that statins improve the outcomes of patients undergoing CABG. The benefits seem to outweigh the potential risks that may accompany usage, both in the preoperative and postoperative period. In the absence of contraindications, essentially all CABG patients are candidates for lifelong statin therapy that ideally should be started before surgery [27]. Propofol was used for 15 hours in a dose of less than 1 mg/kg/h; the risk factor for devlopement of RML associated with propofol infusion syndrome has been identified after useage of doses as a high as 5 mg/kg/h for more than 48 hours [28]. Epinenphrine was used for 240 minutes after coming from CPB, other vasopressors were used in the form norepinhrine, and IABP was not needed as the patient did not initially exhibit low cardiac output state. Our patient developed gram negative septicemia in his course but RML and creatinine peak level were before sepsis started. Rhabdomyolysis can be precipitated by many conditions, and viral as well as bacterial infections are claimed as causing RML [29].

Patient devloped transient impairment of his right ventricular function as evidenced by TTE, in a recent observational study by Olsson *et al.,*[30] the authors found increased risk of heart failure in patients suffered AKI after CABG [30]. We think that multiple factors explained this association in our case including sepsis, prolonged CBP and AKI. Severe RML triggers a cascade with many consequences, including hypovolemia, hypoalbuminemia, anemia, disseminated intravascular coagulation (DIC), hyperkalemia, hypocalcemia, hypercalcemia, hyperphosphatemia, and acute tubular necrosis. It is possible that higher pressures related to increased weight as well as other potential mechanisms related to the metabolic derangement are probably present [31].

When the patient is under sedation it is usually difficult to diagnose RML, usually it requires a high index of clinical suspicion, and early prevention makes sense before later devlopment of renal dysfunction [32]. Hyperkalemia was the alert to start searching for RML. Later our patient devloped AKI necessitating regular hemodialysis which continued for 40 days until the patient regained his renal functions, the likhood of AKI associated with RML may occur with CK level as low as 5000 U/L when there is association of hypovolemia, sepsis and acidosis, AKI associated RML usually carries higher mortality than when RML devloped alone 59% versus 22% respectively [33].

## Conclusions

Here we report on a patient who presented with numerous risk factors for developing rhabdomyolysis following surgical intervention. AKI developed in this patient with prolonged length of ICU stay as well as hospitalization. Early diagnosis is essential for preventing deterioration.

## Consent

Written informed consent was obtained from the patient for publication of this Case Report and any accompanying images. A copy of the written consent is available for review by the Editor-in-Chief of this journal.

## Abbreviations

ACT: Activated clotting time; ASA: American Society of Anesthesiologists; AKI: Acute kidney injury; CABG: Coronary artery bypass graft; CAD: Coronary artery disease; CK: Creatine kinase; CPB: Cardiopulmonary bypass; CTICU: Cardiothoracic surgery intensive care unit; ECG: Electrocardiogram; ETCO2: End tidal carbon dioxide; FFP: Fresh frozen plasma; ID: Internal diameter; LAD: Left anterior descending; LIMA: Left internal mammary artery; LOS: Low cardiac output syndrome; MAC: Minimum alveolar concentration; PO: Per oral; PRBC: Packed red blood cells; PTCA: Percutaneous transluminal coronary angioplasty; RCA: Right coronary artery; RIMA: Right internal mammary artery; RML: Rhabdomyolysis; SPO2: Pulse oximetry; SSDU: Surgical step down unit; TEG: Thromboelastograph; TTE: Trans thoracic echocardiogram; WBC: White blood cells; WHO: World Health Organization.

## Competing interests

The authors declared that they have no competing interests.

## Authors’ contributions

SS, carried out data collection, writing the clinical course. AO, wrote the main manuscript, initial case diagnosis, and submission. AP, as chair of anesthesia, and intensive care provided general support. AA, was the surgeon involved in operating the case, general supervision, and provided general support. All authors read and approved the final manuscript.
